# Supplementary material for: The impact of a wireless audio system on communication in robotic-assisted laparoscopic surgery: A prospective controlled trial
Source: PLoS One. 2020 Jan 10;15(1):e0220214. doi: 10.1371/journal.pone.0220214 (PMC6953850; doi:10.1371/journal.pone.0220214)
Supplement: S1 Table — (DOCX) [file pone.0220214.s001.docx]

Table S1. Questionnaire

|  | **Item** |
| --- | --- |
| 1 | I heard clearly during the case |
| 2 | I had to repeat myself because people didn’t understand/hear my message the first time |
| 3 | Overall I felt that OR team communication was |
| 4 | I would grade my focus and concentration during this case |
| 5 | Steps took longer than necessary because I or others had to repeat/clarify what they were saying |
| 6 | Based on the team’s performance during this case, I would feel perfectly safe being treated here |
| 7 | How successful were you in performing your task? |
| 8 | Team morale during this case was high |
| 9 | I felt comfortable intervening in this procedure when I had concerns about what was occurring |
| 10 | Overall, we worked efficiently as a team |
| 11 | I felt fatigued/exhausted after this case |
| 12 | I was irritated, stressed, or annoyed during this case |
| 13 | How hard did you have to work mentally and physically to accomplish your task? |
| 14 | I was distracted, annoyed, stressed, or bothered by the noise level in the room |
