# Supplementary material for: The impact of a wireless audio system on communication in robotic-assisted laparoscopic surgery: A prospective controlled trial
Source: PLoS One. 2020 Jan 10;15(1):e0220214. doi: 10.1371/journal.pone.0220214 (PMC6953850; doi:10.1371/journal.pone.0220214)
Supplement: S2 Table — (DOCX) [file pone.0220214.s002.docx]

Table S2. The mean score for each statement with and without the headsets, stratified by responder role^a^

| **ITEM\ROLE** | **Attending** | **Anesthesia**^b^ | **Circulator** | **ST** | **Fellow** | **Resident** | **FA** |
| --- | --- | --- | --- | --- | --- | --- | --- |
| **WITH HEADSETS** | | | | | | | |
| Overall | 108.6 ± 3.6 | 113.6 ± 2.3 | 112.3 ± 2.6 | 116.4 ± 2.9 | 117.5 ± 4.6 | 108.1 ± 3.6 | 115.4 ± 5.1 |
| Heard clearly during case* | 8.7 ± 0.4 | 9.2 ± 0.2 | 8.6 ± 0.3 | 8.9 ± 0.3 | 9.3 ± 0.5 | 8.7 ± 0.4 | 9.3 ± 0.6 |
| Needed to repeat | 7.1 ± 0.4 | 7.7 ± 0.3 | 7.5 ± 0.3 | 8.0 ± 0.4 | 8.3 ± 0.5 | 6.7 ± 0.4 | 7.7 ± 0.6 |
| Team communication | 8.4 ± 0.4 | 8.6 ± 0.2 | 8.5 ± 0.3 | 8.7 ± 0.3 | 9.2 ± 0.5 | 8.4 ± 0.4 | 8.8 ± 0.5 |
| Focus† | 8.0 ± 0.3 | 8.0 ± 0.2 | 8.4 ± 0.2 | 8.9 ± 0.3 | 9.0 ± 0.4 | 8.2 ± 0.3 | 8.8 ± 0.5 |
| Steps took longer | 7.6 ± 0.4 | 7.8 ± 0.3 | 7.6 ± 0.3 | 8.1 ± 0.3 | 8.3 ± 0.5 | 7.9 ± 0.4 | 7.6 ± 0.5 |
| Feel safe | 9.3 ± 0.4 | 8.8 ± 0.2 | 8.7 ± 0.2 | 8.6 ± 0.3 | 9.6 ± 0.5 | 8.7 ± 0.3 | 9.1 ± 0.5 |
| Successful in task* | 8.9 ± 0.3 | 9.1 ± 0.2 | 8.7 ± 0.2 | 9.0 ± 0.2 | 9.0 ± 0.4 | 8.3 ± 0.3 | 9.0 ± 0.4 |
| High team morale* | 8.8 ± 0.3 | 9.0 ± 0.2 | 8.8 ± 0.2 | 9.1 ± 0.2 | 8.8 ± 0.3 | 8.5 ± 0.3 | 9.1 ± 0.4 |
| Participation* | 9.1 ± 0.3 | 8.9 ± 0.2 | 8.7 ± 0.2 | 8.6 ± 0.2 | 9.4 ± 0.4 | 8.5 ± 0.3 | 9.3 ± 0.4 |
| Efficient teamwork | 8.7 ± 0.3 | 9.3 ± 0.2 | 8.8 ± 0.2 | 9.2 ± 0.2 | 9.3 ± 0.3 | 8.7 ± 0.3 | 8.9 ± 0.4 |
| Felt exhausted* | 6.5 ± 0.5 | 7.5 ± 0.3 | 7.0 ± 0.3 | 7.6 ± 0.4 | 6.8 ± 0.6 | 6.1 ± 0.5 | 7.4 ± 0.6 |
| Felt stressed/irritated* | 6.3 ± 0.4 | 8.0 ± 0.3 | 7.6 ± 0.3 | 8.2 ± 0.3 | 7.8 ± 0.5 | 7.2 ± 0.4 | 7.6 ± 0.5 |
| Hard work for task | 5.7 ± 0.6 | 5.3 ± 0.3 | 6.3 ± 0.4 | 6.2 ± 0.4 | 6.2 ± 0.7 | 5.5 ± 0.5 | 5.5 ± 0.8 |
| Noise bothered/distracted† | 6.5 ± 0.4 | 7.3 ± 0.3 | 7.6 ± 0.3 | 8.1 ± 0.3 | 8.3 ± 0.5 | 7.5 ± 0.4 | 7.3 ± 0.6 |
| **WITHOUT HEADSETS** | | | | | | | |
| Overall | 98.9 ± 3.6 | 104.3 ± 2.4 | 105.1 ± 2.7 | 104.9 ± 3.0 | 104.1 ± 4.5 | 97.7 ± 3.6 | 95.8 ± 5.0 |
| Heard clearly during case* | 7.1 ± 0.4 | 6.9 ± 0.3 | 7.3 ± 0.3 | 7.2 ± 0.3 | 7.6 ± 0.5 | 7.2 ± 0.4 | 6.9 ± 0.6 |
| Needed to repeat | 5.3 ± 0.4 | 5.9 ± 0.3 | 6.5 ± 0.3 | 6.4 ± 0.4 | 5.9 ± 0.5 | 5.5 ± 0.4 | 5.6 ± 0.6 |
| Team communication | 7.5 ± 0.4 | 7.4 ± 0.2 | 7.6 ± 0.3 | 7.7 ± 0.3 | 8.0 ± 0.5 | 6.8 ± 0.4 | 7.2 ± 0.5 |
| Focus† | 7.9 ± 0.3 | 8.1 ± 0.2 | 8.2 ± 0.3 | 8.2 ± 0.3 | 8.6 ± 0.4 | 7.2 ± 0.3 | 7.7 ± 0.5 |
| Steps took longer | 6.3 ± 0.4 | 6.8 ± 0.3 | 6.5 ± 0.3 | 6.5 ± 0.3 | 6.3 ± 0.5 | 6.3 ± 0.4 | 5.3 ± 0.5 |
| Feel safe | 8.4 ± 0.4 | 8.4 ± 0.2 | 8.2 ± 0.3 | 8.2 ± 0.3 | 9.3 ± 0.5 | 8.0 ± 0.3 | 8.0 ± 0.5 |
| Successful in task* | 8.3 ± 0.3 | 9.0 ± 0.2 | 8.6 ± 0.2 | 8.6 ± 0.2 | 8.7 ± 0.4 | 8.1 ± 0.3 | 8.0 ± 0.4 |
| High team morale* | 8.1 ± 0.3 | 8.8 ± 0.2 | 8.7 ± 0.2 | 8.8 ± 0.2 | 8.6 ± 0.3 | 8.1 ± 0.3 | 8.0 ± 0.4 |
| Participation* | 8.5 ± 0.3 | 8.9 ± 0.2 | 8.6 ± 0.2 | 8.6 ± 0.2 | 9.2 ± 0.4 | 8.1 ± 0.3 | 8.2 ± 0.4 |
| Efficient teamwork | 8.0 ± 0.3 | 8.9 ± 0.2 | 8.5 ± 0.2 | 8.8 ± 0.2 | 8.6 ± 0.3 | 8.2 ± 0.3 | 8.0 ± 0.4 |
| Felt exhausted* | 6.4 ± 0.5 | 6.5 ± 0.3 | 6.8 ± 0.3 | 6.8 ± 0.4 | 6.1 ± 0.6 | 5.9 ± 0.4 | 5.9 ± 0.6 |
| Felt stressed/irritated* | 6.6 ± 0.4 | 7.6 ± 0.3 | 7.5 ± 0.3 | 7.3 ± 0.3 | 6.9 ± 0.5 | 6.7 ± 0.4 | 6.5 ± 0.5 |
| Hard work for task | 4.8 ± 0.6 | 5.0 ± 0.3 | 5.3 ± 0.4 | 5.4 ± 0.4 | 5.7 ± 0.7 | 5.6 ± 0.5 | 5.3 ± 0.8 |
| Noise bothered/distracted† | 6.6 ± 0.4 | 7.1 ± 0.3 | 7.4 ± 0.3 | 6.9 ± 0.4 | 6.4 ± 0.5 | 6.9 ± 0.4 | 6.0 ± 0.6 |

FA, first assistant; ST, surgical technician

*p-value<.05

†p-value<.01

^a^For the full version of each statement please refer to Table S1.

^b^Anesthesia: Anesthesiologist/CRNA (certified registered nurse anesthetists)
